# The Adequacy of Pelvic Lymphadenectomy During Radical Cystectomy for Carcinoma Urinary Bladder: A Narrative Review of Literature

**DOI:** 10.3389/fsurg.2021.687636

**Published:** 2021-06-17

**Authors:** Rahul Jena, Nikita Shrivastava, Aditya Prakash Sharma, Gautam Ram Choudhary, Aneesh Srivastava

**Affiliations:** ^1^Department of Urology, All India Institute of Medical Sciences, Jodhpur, India; ^2^Department of Urology, Postgraduate Institute of Medical Education and Research, Chandigarh, India; ^3^Department of Urology and Renal Transplant, Sanjay Gandhi Postgraduate Institute of Medical Sciences, Lucknow, India

**Keywords:** bladder cancer, pelvic lymphadenectomy, extended lymphadenectomy, pelvic lymph node dissection, super extended pelvic lymphadenectomy

## Abstract

An adequate pelvic lymph node dissection (PLND) is an essential part of radical cystectomy for muscle invasive bladder cancer. However, the definition of what constitutes an adequate PLND is often shrouded in controversy. Various authors have defined different anatomic templates of PLND based on levels of pelvic lymph nodes. Some have suggested other surrogate markers of the adequacy of PLND, namely lymph node count and lymph node density. While individual studies have shown the efficacy and reliability of some of the above markers, none of them have been recommended forthright due to the absence of robust prospective data. The use of non-standardized nomenclature while referring to the above variables has made this matter more complex. Most of older data seems to favor use of extended template of PLND over the standard template. On the other hand, one recent randomized controlled trial (RCT) did not show any benefit of one template over the other in terms of survival benefit, but the study design allowed for a large margin of bias. Therefore, we conducted a systematic search of literature using EMBASE, Medline, and PubMed using PRISMA-P checklist for articles in English Language published over last 20 years. Out of 132 relevant articles, 47 articles were included in the final review. We have reviewed existing literature and guidelines and have attempted to provide a few suggestions toward a uniform nomenclature for the various anatomical descriptions and the extent of PLND done while doing a radical cystectomy. The results of another large RCT (SWOG S1011) are awaited and until we have a definitive evidence, we should adhere to these suggestions as much as possible and deal with each patient on a case to case basis.

## Introduction

Each year, more than 400,000 patients worldwide are diagnosed with bladder cancer of which ~30% are muscle invasive (1). Radical cystectomy (RC) with bilateral pelvic lymph node dissection (PLND) is the standard of care for recurrent high risk non muscle invasive bladder cancer (NMIBC) and muscle invasive bladder cancer (MIBC) (2, 3). Preoperative cross-sectional imaging, with a sensitivity of about 52% for positive pelvic lymph nodes, often leads to significant under staging (4–6). A thorough bilateral PLND therefore increases the staging procedure's accuracy and provides a probable survival benefit in patients of MIBC irrespective of the nodal involvement (7, 8). Now the question arises as to how should one assess the adequacy of PLND? Whether it is the levels of pelvic lymph nodes removed, the anatomical template of dissection followed, the lymph node count or the lymph node density remains a bone of contention? Adding to this confusion, is the frequent use of non-standardized nomenclature in denoting the extents of PLND across various studies. Therefore, we attempted to review the existing literature related to the levels of pelvic lymph nodes and the various templates of PLND defined by different authors to bring clarity to this issue. We also suggest certain points which can bring a uniformity to this procedure and thus facilitate better reporting of the outcomes of PLND for MIBC.

## Methodology

### Search Strategy and Inclusion Criteria

The systematic literature search was done for relevant papers, by two authors RJ and APS, in various electronic databases as follows. The following keywords with the operators from the years 2001 to 2020 were used: (“lymph node^*^” OR standard OR extended OR lymphadenectomy) AND ((“bladder cancer” OR “bladder carcinoma” OR urothelial) AND cancer OR “urothelial carcinoma of the bladder”) AND radical AND cystectomy for searching EMBASE/Medline and the following keywords were used for searching PubMed - (lymphadenectomy) OR (Standard) OR (extended) OR (lymph node) AND ((bladder cancer) OR (urothelial cancer) OR (bladder carcinoma) OR (urothelial carcinoma)) AND (radical cystectomy). The conference abstracts, conference papers, conference review, erratum, and notes were removed and the search results were filtered to include the articles only in English Language.

### Results

**Figure 1:**
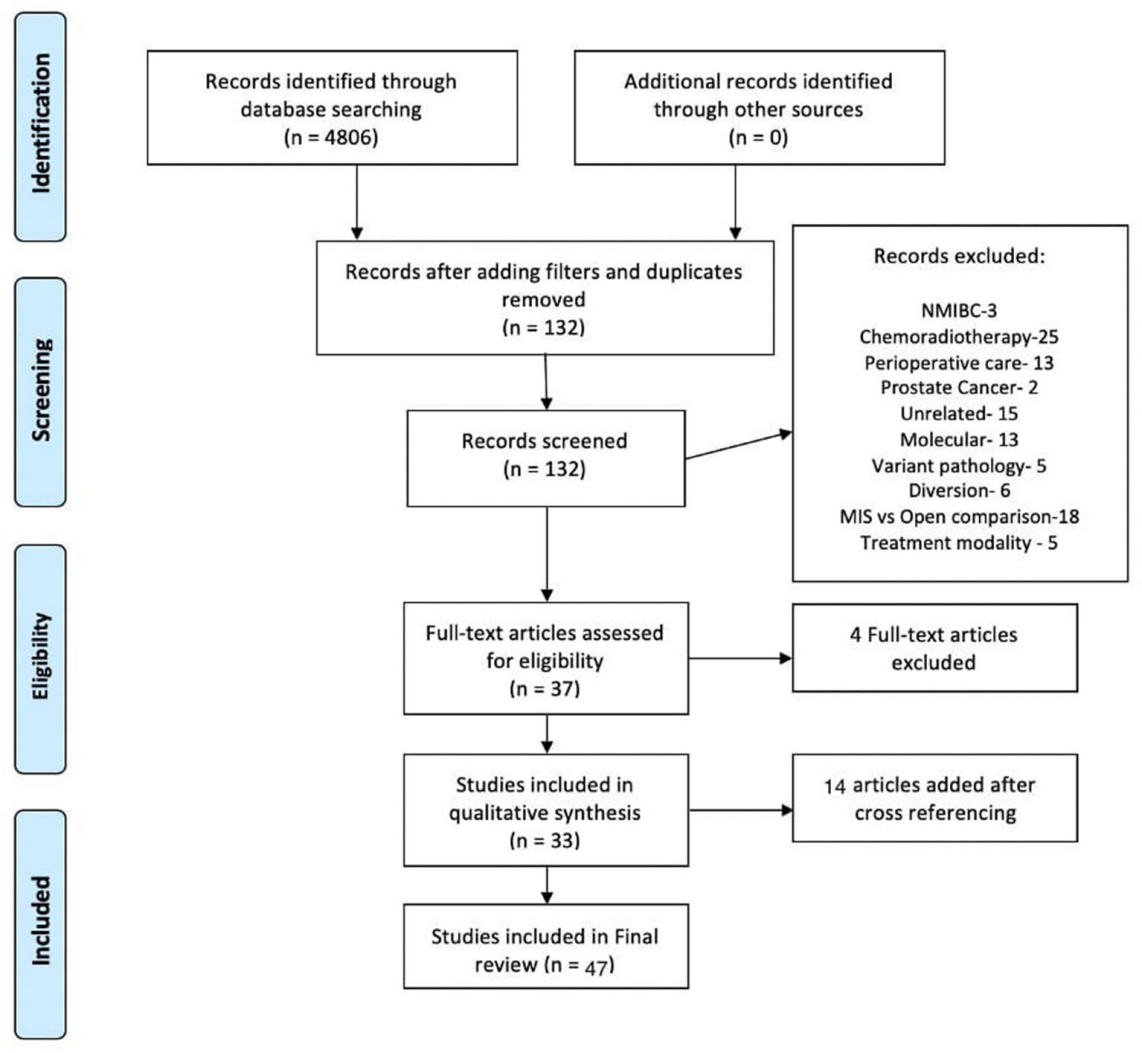
Flowchart showing search strategy and selection of articles for review.

Initial search yielded 4,604 articles from EMBASE and 202 results from PUBMED. After applying exclusion criteria, this narrowed the number of articles to 98 from Embase. After removing the duplicates a total of 132 articles (EMBASE+PUBMED) were assessed by two authors (APS and RJ) independently. The reference list was searched for more relevant articles. Total of 47 articles were selected for review of literature (Table 1). The selection process is outlined in Figure 1. The current narrative review is based on these 47 included articles.

**Table 1:** Details of articles selected for review of literature.

**No**.	**References**	**Year**	**Conclusion**
1	Funt and Rosenberg (1)	2017	The standard of care for muscle invasive bladder cancer is neoadjuvant cisplatin based chemotherapy followed by radical cystectomy and bilateral pelvic lymphadenectomy.
2	Buscarini et al. (2)	2007	Extended pelvic lymph node dissection during radical cystectomy provides diagnostic and therapeutic benefit on muscle invasive carcinoma bladder.
3	Sung and Lerner (3)	2020	The first randomized phase III trial did not show benefit of extended pelvic lymphadenectomy. However, there are many potential shortcomings of this trial. The results of the SWOG 1011 trial should be able to give us a better idea about the benefits of an extended template of dissection.
4	Papalia et al. (4)	2012	Diffusion weighted MRI can differentiate between metastatic and non-metastatic pelvic lymph nodes in patients with high grade bladder cancer.
5	Crozier et al. (5)	2019	PET-CT and MRI are more sensitive than CT scan for detection of positive lymph nodes in bladder cancer prior to cystectomy.
6	Jeong et al. (6)	2015	Combined PET-CT does not have increased sensitivity compared to CT alone for the detection of positive pelvic lymph nodes in patients of bladder cancer prior to radical cystectomy.
7	Bruins et al. (7)	2014	Any pelvic lymph node dissection is better than no pelvic lymph node dissection. Extended dissection seems to be more advantageous than standard dissection. However super extended dissection doesn't provide additional therapeutic or diagnostic benefits.
8	Suttman et al. (8)	2007	Retrospective studies point out that while the benefit of a bilateral pelvic lymphadenectomy during radical cystectomy is unquestionable.
9	Cattaneo et al. (9)	2018	Extended pelvic lymph node dissection provides optimal diagnostic and therapeutic benefit in patients undergoing radical cystectomy for muscle invasive bladder cancer.
10	Abol-Enein et al. (10)	2004	The internal iliac and obturator group of lymph nodes are the sentinel group for bladder cancer. Bilateral dissection of these areas is mandatory. Negative nodes here mean that more proximal dissection is not necessary.
11	Bochner et al. (11)	2004	Extended template pelvic lymph node dissection had a significantly higher lymph node lymph node yield compared to standard dissection even though it doesn't provide any staging advantage.
12	Roth et al. (12)	2010	Standard template of pelvic lymph node dissection removes only 50% of all lymph nodes in the primary landing sites of bladder cancer while extended lymphadenectomy removes about 90%.
13	Leissner et al. (13)	2004	Extended radical cystectomy should be the standard of care in all patients of radical cystectomy. No sentinel lymph nodal area was identified.
14	Perera et al. (14)	2018	Extended pelvic lymphadenectomy provides optimal recurrence free and cancer specific survival. Super extended template provides no actual benefit. Increased lymph node yields provides improved oncological outcomes in patients with both node positive or node negative disease.
15	Tarin et al. (15)	2012	Pathological involvement of the common iliac lymph node is not associated with a worse outcome compared to the primary nodal basin disease, thus promoting the inclusion of this group in the primary pathological staging of bladder cancer during radical cystectomy. However number of positive lymph nodes was an independent predictor of poor outcomes.
16	Hwang et al. (16)	2019	Extended pelvic lymphadenectomy may reduce the risk of death from any cause in patients undergoing radical cystectomy for bladder cancer over time compared to standard pelvic lymphadenectomy. However there is a possibility of no effect.
17	Sundi et al. (17)	2014	Extended pelvic lymphadenectomy seems to be adequate for staging and cancer related outcomes. However, the super extended template may be associated with greater morbidity. Risk based approach should be followed to determine template of dissection in each patient.
18	Dorin et al. (18)	2011	Extended pelvic lymphadenectomy with meticulous dissection is more important that total lymph nodal count to achieve optimal oncological outcomes because lymph node metastases outside the boundaries of the standard template are common.
19	Dhar et al. (19)	2008	Extended pelvic lymph node dissection during radical cystectomy allows for more accurate staging and improved survival in patients with node positive and non-organ confined disease.
20	Li et al. (20)	2016	Greater number of dissected lymph nodes are associated with better survival advantages in patients of bladder cancer. Number of dissected lymph nodes could be an independent prognostic factor.
21	Bi et al. (21)	2014	Extended pelvic lymphadenectomy provides better recurrence free survival compared to standard lymphadenectomy in patients with both pathologically positive and negative pelvic lymph nodes.
22	Mandel et al. (22)	2014	Extended pelvic lymphadenectomy has better oncological outcomes and is not associated with greater perioperative mortality or higher complication rates.
23	Wang et al. (23)	2019	Extended pelvic lymphadenectomy has better recurrence free survival and disease specific survival in bladder cancer and is not associated with more postoperative complications compared to non-extended lymphadenectomy.
24	Zehnder et al. (24)	2011	Meticulous extended lymphadenectomy with emphasis on skeletonization of the pelvic vessels has shown to be similar to super extended lymphadenectomy in terms of oncological outcomes. Certain groups with suspicious lymph nodes even after neoadjuvant therapy may need more extensive dissections.
25	Møller et al. (25)	2016	Super extended lymphadenectomy may benefit only a small subgroup of patients with non-organ confined disease without macrometastases and is not beneficial in the general set of patients.
26	Holmer et al. (26)	2009	Extended lymph node dissection seems to have improved time to recurrence and survival, especially in patients with non-organ confined disease.
27	Simone et al. (27)	2013	Extended pelvic lymphadenectomy has significant staging accuracy and survival benefit for bladder cancer across all stage groups.
28	Abdi et al. (28)	2016	Extended pelvic lymphadenectomy appeared to reduce the risk of local recurrence but had no effect on overall survival. It was associated with higher blood loss but similar rates of complications.
29	Hugen et al. (29)	2010	Lymphovascular invasion, perineural invasion and lymph node yield <14 are independent risk factors for bladder cancer recurrence in patients with node negative bladder cancer.
30	Muilwijk et al. (30)	2018	Super extended lymph node dissection has no advantage compared to standard template. However by using a super extended template, we identify 2% more patients as node positive, which would have been falsely diagnosed as node negative using the standard template and resect 35% more positive LNs, which would have been left behind by standard template lymphadenectomy, with a limited increase in morbidity.
31	Gschwend et al. (31)	2019	Lymphadenectomy up to the inferior mesenteric artery failed to show any significant advantage over the standard lymph node dissection in terms of recurrence free survival, cancer specific survival or overall survival.
32	Lerner et al. (32)	2019	Editorial commentary on the LEA trial (31). The study was underpowered to detected smaller benefits that can be attributed to super extended dissection compared to the standard template because of its sample size. Also, since the survival curves showed some divergence toward the end of the follow up period, longer follow up is necessary to get further insights. Also the study was not designed to prove that the limited lymphadenectomy is not inferior to the extended lymphadenectomy.
33	Josephson et al. (33)	2005	Extended template of pelvic lymph node dissection provides greater therapeutic and diagnostic benefit.
34	Boström et al. (34)	2020	Identified clinical markers of morbidity, mortality and survival in patients of bladder cancer treated with radical cystectomy, of which extra nodal extension conferred a poor prognosis.
35	Chou et al. (35)	2016	Extended dissection may confer survival and recurrence free advantages. Neoadjuvant cisplatin based chemotherapy appears to decrease mortality compared to radical cystectomy alone.
36	May et al. (36)	2011	Removal of higher number of lymph nodes is associated with improved oncological outcomes. Use of an extended template of dissection along with assessment of lymphovascular invasion is essential in stratifying patients into risk groups and to identify those who might benefit from adjuvant therapy.
37	Morgan et al. (37)	2012	Lymph node count at radical cystectomy is a predictor of overall survival and disease specific survival in patients with pathologically node negative disease but not in patients with pathologically positive lymph nodes.
38	Herr et al. (38)	2002	A greater number of lymph nodes is associated with a better staging and impact patient outcomes. Along with therapeutic and staging benefits it also helps identify patients who would benefit from adjuvant therapy.
39	VAN Bruwaene et al. (39)	2016	Predictors like total number of lymph nodes, number of positive lymph nodes, lymph node density and presence of extra nodal extension along with tumor characteristics like T stage and histology and neoadjuvant chemotherapy should be incorporated into normograms used for prognosticating patients who have undergone radical cystectomy.
40	Matsumoto et al. (40)	2015	Extended pelvic lymph node dissection helps in improving prognosis by eliminating micrometastases.
41	Cha et al. (41)	2015	There is no concrete evidence to favour extended pelvic lymphadenectomy over standard lymphadenectomy alone.
42	Capitanio et al. (42)	2009	Removing a minimum of 25 lymph nodes confers a 75% probability of detecting lymph node metastases and removing atleast 45 nodes gives a 90% probability. 15 lymph nodes have 50% probability and thus the goal is that atleast 25 lymph nodes should be removed during radical cystectomy.
43	Koppie et al. (43)	2006	There is no minimum lymph nodal count that can optimize outcomes after radical cystectomy. However increasing nodal yield is associated with increasing probability of survival. This highlights that extended lymphadenectomy should be done to improve outcomes.
44	Ku et al. (44)	2015	Lymph node density is an independent predictor of clinical outcome in lymph node positive patients after radical cystectomy.
45	Lee et al. (45)	2012	Lymph node density is an useful tool for risk stratifying patients after radical cystectomy and higher lymph node density has poorer disease specific survival in node positive patients.
46	Kondo et al. (46)	2012	Extended lymph node dissection improves oncological outcomes after radical cystectomy. Lymph node density is an important predictor of overall survival in node positive patients.
47	Ahn et al. (47)	2015	Extracapsular extension is an important prognostic factor for node positive bladder cancer.

### Anatomy of Lymphatic Drainage of Bladder

**Figure 2:**
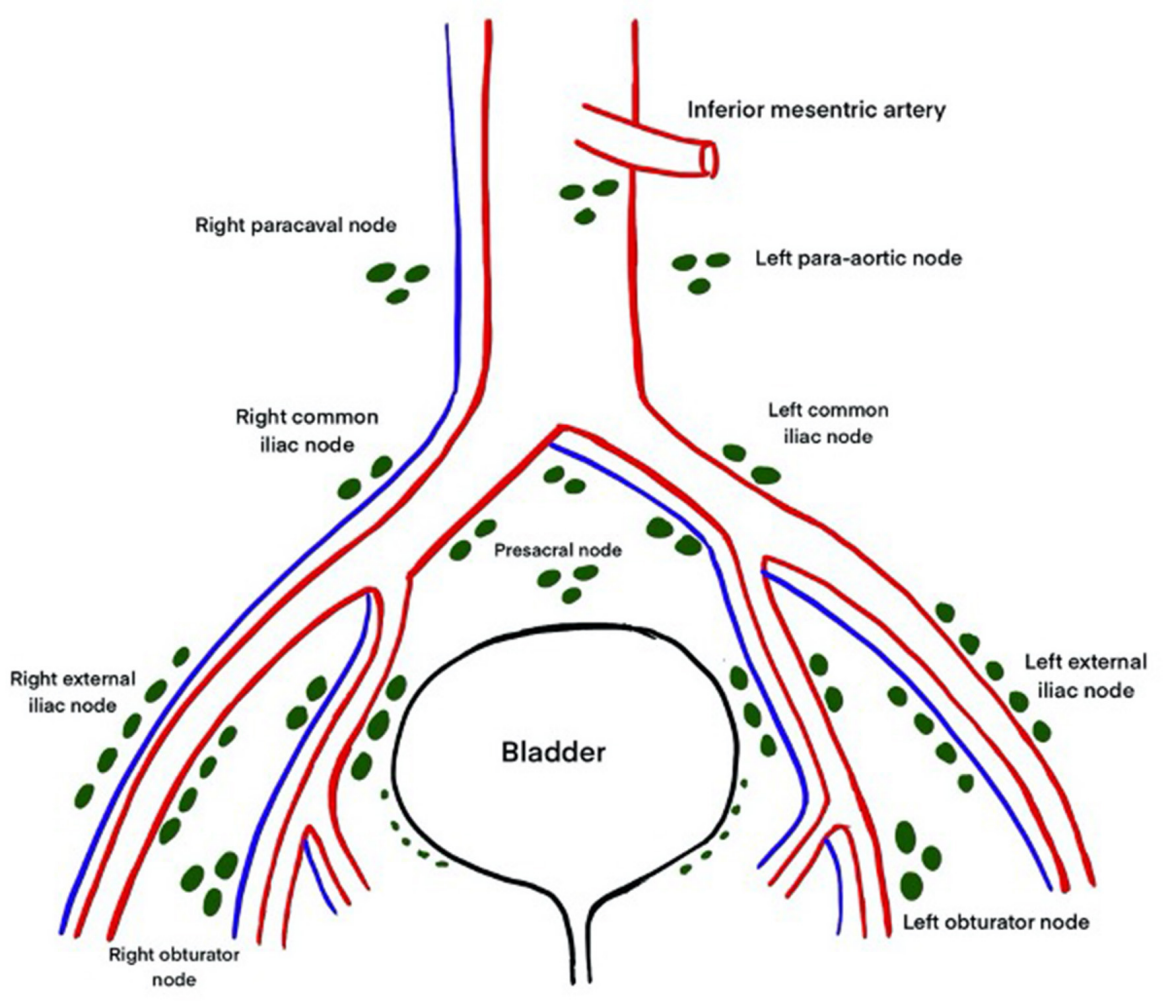
Anatomy of lymphatic drainage of the bladder.

The primary drainage site for the bladder consists of the external iliac, internal iliac, obturator, and presacral lymph nodes. The secondary drainage goes to the common iliac, para-aortic, inter-aortocaval, and para-caval lymph nodes (Figure 2) (9). Bilaterality of lymph nodal spread has been demonstrated in up to 39% of patients. It has been confirmed using single- photon emission computed tomography (SPECT) and an intraoperative γ-probe after injection with a technetium nano-colloid, which has also shown that up to 52% of nodes may lie outside the true pelvis (10–12). Leissner et al. (13) have identified 12 anatomical sites with variable probability of metastatic deposits, with the obturator groups being the commonest involved site. In this study, 6.9% patients had metastases in the regions above the common iliac bifurcation and 2.9% had metastases in the inter-aortocaval and precaval regions. Many subsequent studies have shown that up to 41% of positive lymph nodes lie above the bifurcation of the common iliac arteries (14). In 591 patients, Tarin et al. (15) reported lymph node involvement in 1194 patients (19%). Of these, seven patients (6%) had no positive lymph nodes within the true pelvis (skip lesions). Since skip lesions are known to be very rare, this phenomenon may be the result of missed positive lymph nodes in the true pelvis or of a specimen-labeling error. But a few things are clear. First, PLND should be bilateral since drainage is bilateral. Next, a limited PLND template has a small but significant chance of missing positive nodes lying outside the true pelvis. However, whether wider dissection necessarily translates into oncological advantage needs to be seen.

### Anatomical Variables Used to Assess Adequacy of Pelvic Lymph Node Dissection

#### Levels of Pelvic Lymph Node Dissection

**Figure 3:**
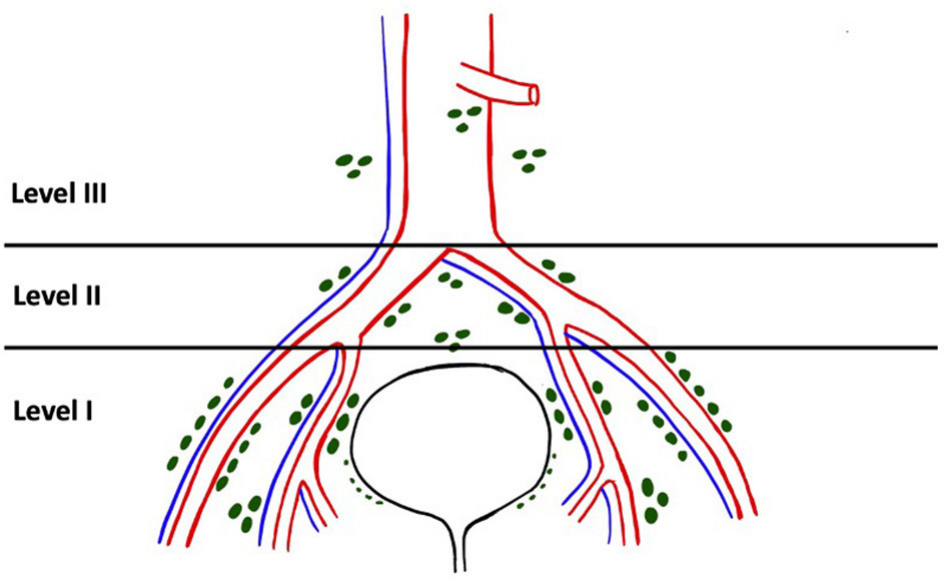
Levels of lymph node dissection during radical cystectomy as described by Leissner et al.

In their paper in 2004, Leissner et al. (13) proposed a 3-tier classification system for the extent of PLND during radical cystectomy (Figure 3). The anatomical sites and their boundaries have been described in Table 2. In the more contemporary studies, this system of denoting extent of PLND has been used sparingly compared to the anatomical templates discussed in the next section (16).

**Table 2:** Description of anatomical fields for extended PLND by Leissner et al.

**Anatomical site**	**Boundaries (cranial—caudal—medial—lateral)**
Right para-caval	Level of inferior mesenteric artery—aortic bifurcation—midline of vena cava—right ureter
Inter aortocaval	Level of inferior mesenteric artery—aortic bifurcation—midline of vena cava—midline of aorta
Left paraaortic	Level of inferior mesenteric artery—aortic bifurcation—midline of aorta—left ureter
Lateral to right common iliac artery	Aortic bifurcation—bifurcation of internal and external iliac arteries—midline of common iliac artery—psoas muscle
Lateral to left common iliac artery	Aortic bifurcation—bifurcation of internal and external iliac arteries—midline of common iliac artery—psoas muscle
Lateral to right external iliac artery	Bifurcation of internal and external iliac arteries—pelvic floor—midline of external iliac artery—genitofemoral nerve
Lateral to left external iliac artery	Bifurcation of internal and external iliac arteries—pelvic floor—midline of external iliac artery—genitofemoral nerve
Pre-sacral	Triangle between midline of the common iliac arteries—bifurcation of internal and external iliac arteries, dorsal border is sacrum
Right obturator space	Bifurcation of internal and external iliac arteries—pelvic floor—obturator nerve—midline of external iliac artery
Left obturator space	Bifurcation of internal and external iliac arteries—pelvic floor—obturator nerve—midline of external iliac artery
Right deep obturator space	Origin of the obturator nerve—pelvic floor—bladder wall—pelvic side wall
Left deep obturator space	Origin of the obturator nerve—pelvic floor—bladder wall—pelvic side wall

#### Templates of Pelvic Lymph Node Dissection

**Figure 4:**
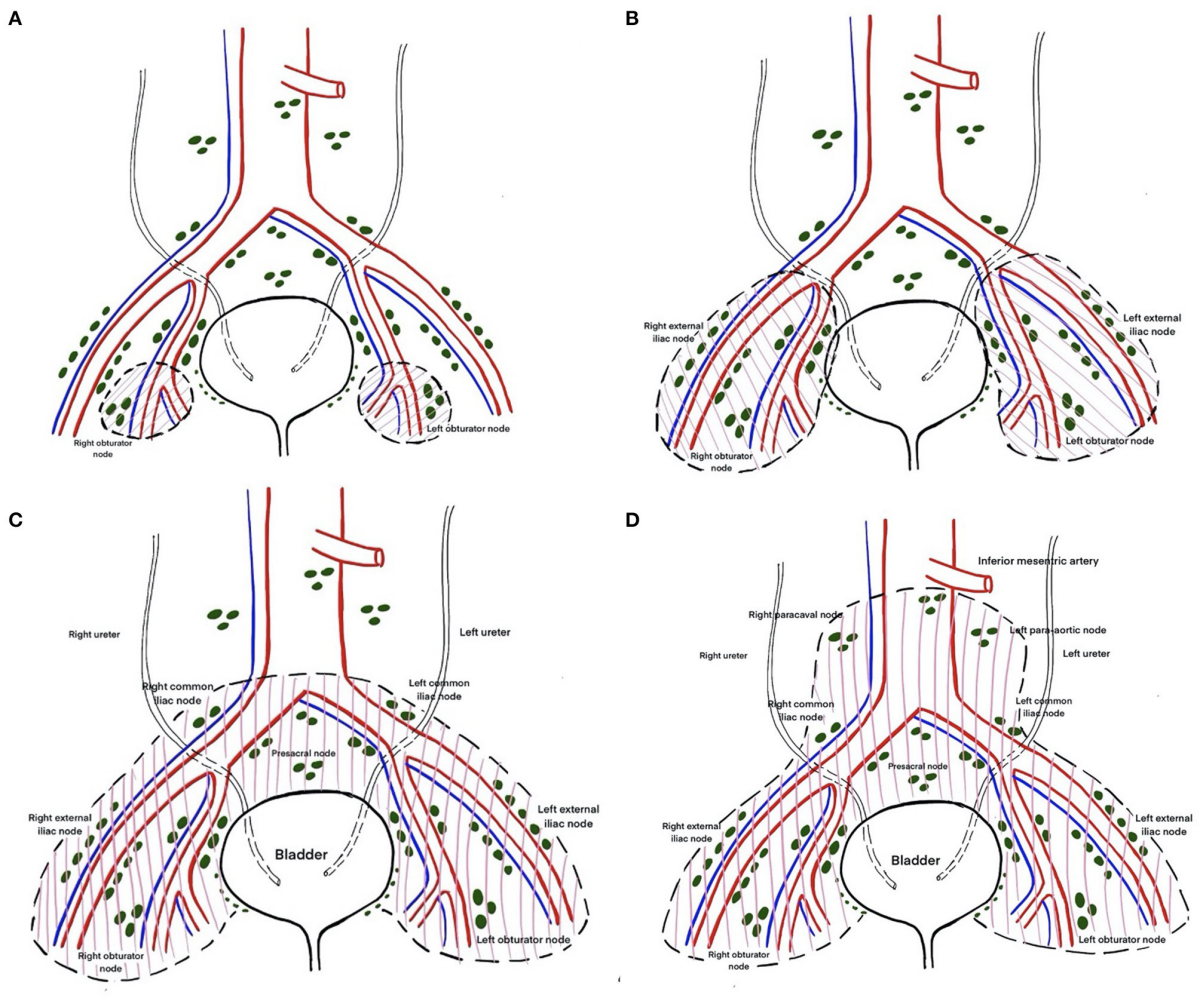
**(A)** Limited lymph node dissection including only bilateral obturator and perivesical lymph nodes. **(B)** Standard template of lymph node dissection including lymph nodes of the external and internal iliac groups, up to bifurcation of the common iliac artery. **(C)** Extended template of lymph node dissection including lymph nodes up to the aortic bifurcation. **(D)** Super extended lymph node dissection including lymph nodes above the aortic bifurcation below the origin of the inferior mesenteric artery.

The EAU Working Group on MIBC proposed the following nomenclature for the anatomical templates used in PLND based on the recommendations of an expert panel: *limited, standard, extended*, and *super-extended PLND*. The definition of these terms has been rather inconsistent and studies have often termed anything less than an extended template as a limited template. Limited PLND typically includes dissection restricted to the bilateral obturator fossae (Figure 4A) (14). Boundaries of standard PLND include the common iliac bifurcation cranially and the inguinal ligament caudally. Laterally the boundaries are the genitofemoral nerve and medially it is the bladder wall. This template typically includes the distal common iliac, the external iliac, the obturator and the internal iliac lymph nodes bilaterally (Figure 4B) (14, 17). In addition to all the lymph nodes removed in the standard template, extended dissection entails removing the presacral nodes and all the nodes between the aortic and the common iliac bifurcations (Figure 4C) (14, 17). Super-extended PLND template involves removal of all the nodal tissue caudal to the base of the inferior mesenteric artery (Figure 4D) (14).

### Adequate Pelvic Lymph Node Dissection

#### Limited vs. Extended PLND

Limited PLND has been shown to miss about 50% of positive lymph nodes in muscle invasive bladder cancer (12). The rationale behind performing a standard template PLND is that 90–95% of all node positive patients can be identified (13, 18). Dhar et al. (19) retrospectively compared limited PLND to extended PLND in 658 patients across 2 centers. It is important to point out here that what the authors describe as a limited PLND in their report is actually a standard template PLND we have described earlier. This discrepancy is an example of how a non-standardized system of nomenclature is fraught with confusion. In this study, 26% node positive patients were identified in the extended template cohort compared to 13% in the limited PLND cohort. Five-years recurrence free survival (RFS) was 23 vs. 57% (*p* < 0.0001), and overall survival (OS) was 26 vs. 46% (*p* = 0.0021), in favor of the extended LND group. For node positive patients the 5-year relapse-free survival and overall survival were both 7% for a limited dissection compared with 35 and 34% for patients undergoing extended LND, respectively (*p* < 0.0001). The authors concluded that standard PLND is associated with suboptimal staging and poorer outcomes for node positive and node negative disease with comparable pT stage and a higher rate of local progression, as summarized in Table 2.

In a meta-analysis by Li et al. (20), a greater extent of LND during RC had statistically significant advantages in OS, CSS and RFS, corresponding to reduced risks of 28, 34, and 36%, respectively. Bi et al. (21) showed that extended PLND was associated with improved RFS (HR = 0.66, 95% CI: 0.56–0.78, and *p* < 0.001) (21). The benefits of extended PLND were present in node-negative disease (HR = 0.68, 95% CI: 0.51–0.90, and *p* = 0.007), node-positive disease (HR = 0.58, 95% CI: 0.47–0.72, and *p* < 0.001), and pT3–4 disease (HR = 0.61, 95% CI: 0.52–0.73, and *p* < 0.001). Similar results were obtained from other meta-analyses (22, 23). A source of bias in the above studies was that the demographics of the patient populations were different and PLND was performed at the clinician's discretion. In a recent systematic review, the influence of PLND on perioperative and oncologic outcomes in patients undergoing RC for MIBC was assessed (7). Due to large heterogeneity between the studies the original meta-analysis planned by the authors was not possible. But the final results showed that any PLND is better than no PLND and extended template might improve oncologic outcomes compared to standard PLND. However, the benefit of super-extended templates is unlikely when compared to extended template PLND.

#### Extended vs. Super-Extended PLND

Several groups published their results comparing extended PLND to super-extended PLND as mentioned in Table 2. Zehnder et al. (24) showed that super-extended PLND was not associated with a significantly improved 5-year RFS or OS even when stratified by node positivity. Møller et al. (25) did a similar comparison in 578 patients and reported no significant differences in RFS in the extended and super-extended groups. A trend toward increased RFS was seen in the >pT3 group in the super-extended cohort but was statistically insignificant. The younger age of patients in the super-extended cohort was also a potential source of bias. In light of the above evidence, super extended PLND appears to have no oncological benefit over extended PLND. This is probably because of the fact that metastatic spread beyond the anatomical pelvis could increase the risk of metastatic disease and further nodal deposits beyond the boundaries of the super-extended template.

#### Other Studies Comparing Various Lymph Node Dissection Templates

Holmer et al. (26) analyzed 69 and 101 patients under-going limited PLND (perivesical and obturator nodes) and standard PLND (limited regions plus the internal, external and common iliac nodes, and presacral nodes), respectively. They found no significant difference in DSS between the two groups. However, patients with pT3-4a disease were more common in the standard PLND group than in the limited one. Multivariate analysis revealed that there was significantly improved survival in the patients who underwent standard PLND, as shown in Table 3. Simone et al. (27) supported that extended PLND has both staging and therapeutic roles reporting better oncologic outcomes of patients who underwent extended PLND than standard PLND. They showed that patients who underwent an extended PLND had a significant improvement of disease-free survival (DFS) (HR = 1.96, 95% CI: 1.56–2.47, and *P* < 0.001) and CSS (HR = 1.76, 95% CI: 1.36–2.99, and *P* < 0.001) probabilities compared to s-PLND. Thus, they described a therapeutic result of extending PLND from the iliac bifurcation up to the aortic bifurcation. In a study by Abdi et al., extended PLND appeared to reduce the risk of local recurrence, but was not an independent predictor of overall survival (28). Extended PLND was associated with greater blood loss than s-PLND, but not with other perioperative complications. In contrast, Hugen et al. (29) compared standard and extended lymph node dissection and found no difference in 5-year recurrence free survival when stratified by node yield using the Kaplan–Meier method (*P* = 0.138). Table 3 summarizes the conclusion drawn by various studies on the adequacy of pelvic lymph node dissection extent.

**Table 3:** Comparison of lymph node yield in different PLND templates in various studies.

**S.NO**.	**References**	**Year**	**Type of LND**		**Number of cases**		**Median lymph node yield**		**Primary end point**	**Conclusion**
			**Control group**	**Intervention group**	**Control group**	**Intervention group**	**Control group**	**Intervention group**		
1	Bochner et al. (11)	2004	sLND	eLND	72	72	8	22	Staging advantage	No staging advantage was observed in eLND group as compared to sLND
2	Dhar et al. (19)	2008	sLND	eLND	336	322	12	22	RFS, OS	RFS 23 vs. 57% (*p* < 0.0001), OS 26 vs. 46% (*p* = 0.0021), in favor of the extended LND group
3	Zehnder et al. (24)	2011	eLND	seLND	405	554	22	38	RFS, OS	sePLND not associated with a significantly improved 5-year RFS or OS when stratified by node positivity
4	Holmer et al. (26)	2009	lLND	sLND	69	101	8	37	DSS	No significant difference in DSS
5	Simone et al. (27)	2012	sLND	eLND	584	349	18	29	DFS, CSS	e-PLND group had a significant improvement of DFS (*P* < 0.001) and CSS (*P* < 0.001) compared to s-PLND
6	Abdi et al. (28)	2016	sLND	eLND	105	105	9	21	RFS, OS	ePLND associated with a better local recurrence free survival (HR = 0.63, *P* = 0.005), but not an independent predictor of overall survival (HR = 1.06, *P* = 0.84)
7	Hugen et al. (29)	2010	sLND	eLND	206	54	9	46	RFS	No difference in 5-year RFS when stratified by node yield
8	Gschwend et al. (31)	2019	lLND	eLND	203	198	19	31	RFS, OS, CSS	eLND failed to show superiority over lLND with regard to RFS (5-year RFS 65 vs. 59%; hazard ratio [HR] = 0.84 [95% confidence interval 0.58–1.22]; *p* = 0.36), CSS (5-year CSS 76 vs. 65%; HR = 0.70; *p* = 0.10), and OS (5-year OS 59 vs. 50%; HR = 0.78; *p* = 0.12)

*LND, lymph node dissection; lLND, limited LND; sLND, standard LND; eLND, extended LND; seLND, super-extended LND; RFS, recurrence free survival; OS, overall survival; DSS, disease specific survival; DFS, disease free survival; CSS, cancer specific survival*.

#### Lessons From the LEA and SWOG S1011 Trials (Table 3)

The above literature supporting extended PLND was somewhat challenged by the findings of the LEA trial, which is the first prospective randomized phase III trial comparing standard PLND with super-extended PLND (31). Extended LND (*n* = 198) failed to show a significant advantage over standard LND (*n* = 203) for RFS [5-year RFS 65 vs. 59%; hazard ratio 0.84; 95% confidence interval (CI): 0.58–1.22], cancer-specific survival (CSS) (5-year CSS 76 vs. 65%; HR 0.70; 95% CI 0.46–1.07) and OS (5-year OS 59 vs. 50%; HR 0.78; 95% CI: 0.57–1.07). However, a significant number of confounding factors are evident in this study. None of the patients received neoadjuvant chemotherapy. The relatively high percentage (14%) of pT1 disease could have limited the results' strength since more extensive PLND usually benefits those with more advanced disease. Post-operative chemotherapy was given at the discretion of the physician. Also, the relatively high percentage of positive surgical margins, 8.9% in the limited and 8.6% in the extended arm, raise questions about the adequacy of RC and if this could have influenced the OS and RFS. Lastly, this study was not powered to demonstrate the non-inferiority of standard PLND to super-extended PLND.

However, this trial has given us some valuable pointers. Firstly, 11% patients had positive lymph nodes located outside the standard PLND template in the super-extended group. If these patients had undergone a standard PLND, 2% would have had a false diagnosis of node negative disease. Also, of the total number of identified lymph nodes, 35% were solely in the super-extended template and would have been missed in a standard PLND. So, by using a super-extended, the authors identified 2% more patients as node positive and resected 35% more positive LNs, which would have been left behind using a standard PLND, with a limited increase in morbidity. In addition, the survival curves show some clinically significant but statistically insignificant divergence in respect to all the 3 endpoints and longer follow up is necessary. The results of the SWOG S1011 trial are awaited (32). This trial has a similar study design to the LEA trial, but it excludes patients with pT1 disease. Also 56% of patients in this trial received neoadjuvant therapy, hence it is more representative of the real world scenario. The accrual is complete and the estimated completion date is August 2022. Table 4 compares chief characteristics of both of these trials.

**Table 4:** Comparison of the LEA and SWOG-1011 trial (30).

**Characteristics**	**LEA**	**SWOG-1011**
Identifier	NCT01215071	NCT01224665
Status	Completed	Ongoing
Comparing	sLND vs. seLND	sLND vs. seLND
Tumor stage	T1–T4a	T2–T4a
Primary endpoint	RFS at 5 years	RFS at 3 years

### Significance of Lymph Node Count, Lymph Node Density, and Extra-Nodal Extension

Lymph node count from a dissected specimen is influenced by various factors like method of lymph node submission (en-bloc vs. separate packets and the number of packets sent), surgical technique, and variability in the pathologic practices and reporting standards, along with inter individual variability in retrieving lymph nodes from the same template (33). Lymph node density refers to the ratio of positive lymph nodes on histopathology to the total number of nodes removed (29). Extra-nodal extension of tumor in an involved lymph node refers to the growth of a nodal cancer metastasis beyond the confines of the capsule of a lymph node into the adjacent tissues (34).

Multiple authors have reported the decreased probability of cancer death with increased number of lymph nodes harvested (35–39). The mechanism of this benefit is probably the removal of undetected micro-metastases, particularly in the setting of neoadjuvant or adjuvant therapies (40). Confounding factors like patient, surgeon, or institutional factors might also contribute to improved outcomes (41). Capitanio et al. (42) evaluated the probability of detecting node positive disease in a multi-institutional cohort of 731 patients based on total number of lymph nodes removed. 23.8% patients had positive nodes. Using receiver operating characteristic (ROC) curves, the authors predicted a 75% chance of identifying one or more lymph node metastases if 25 nodes were removed which improved to 90% with 45 nodes and decreased to 50% if 15–25 nodes were removed. So, a 25-node minimum was a reasonable cut-off to adequately stage and detect lymph node metastasis. In a retrospective analysis by Koppie et al. (43) from the MSKCC group, multivariate analysis showed that that increased lymph node counts did not correlate with increased survival above a count of 23. However, none of the authors could identify a continuous group of lymph nodes to be an independent predictor of cancer specific survival.

**Figure 5:**
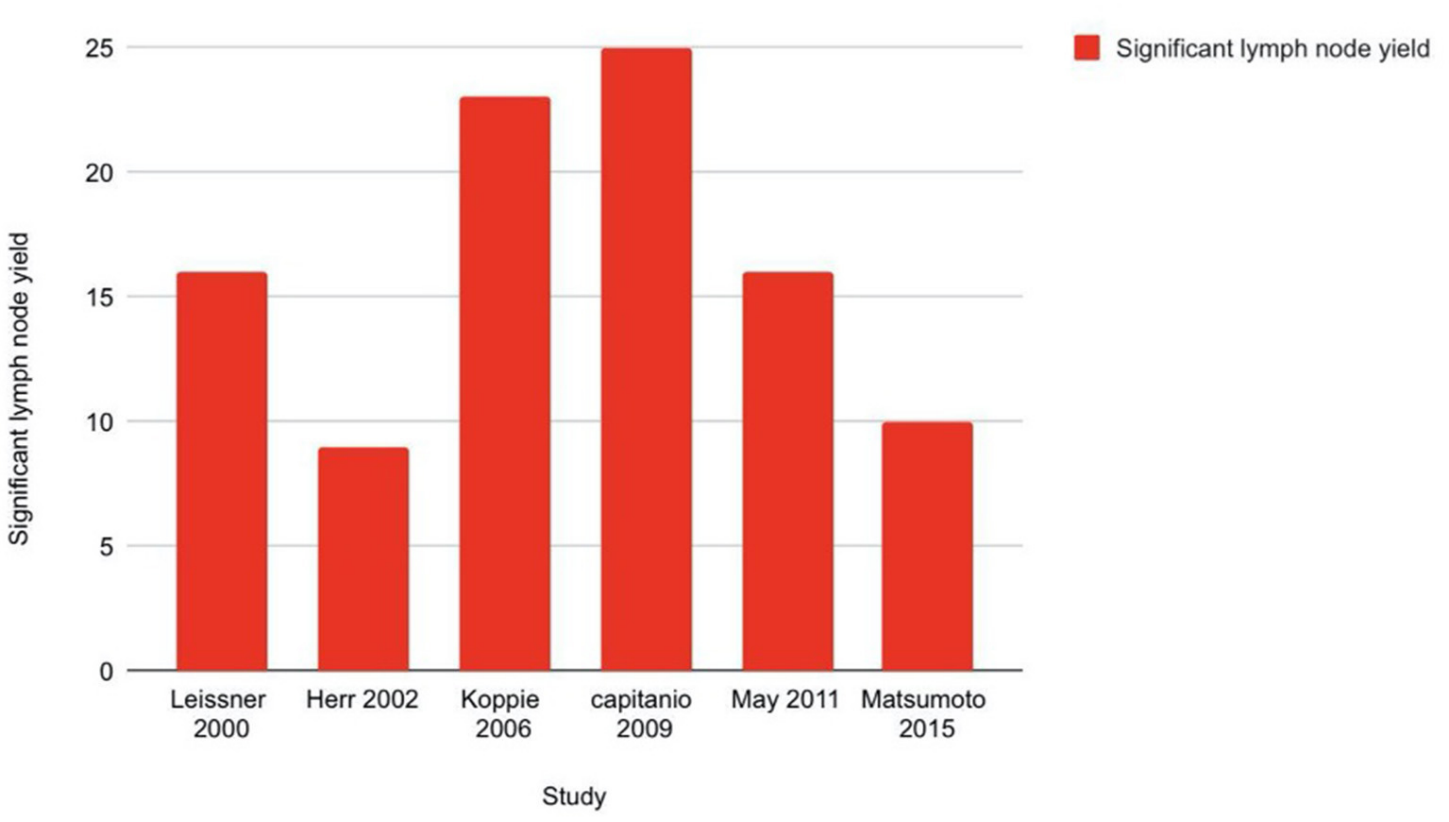
Bar diagram showing minimum lymph node yield to be predictive of outcome of urinary bladder cancer.

Increased lymph node density >20% decreases OS to <10% (41, 44–46). Extra-nodal extension of tumor is also a bad prognostic factor. A meta-analysis of 1,893 patients showed that it considerably correlated with reduced RFS (HR = 1.56, 95% CI: 1.13–2.14) and cancer-specific survival (HR = 1.60, 95% CI: 1.29–1.99) but not OS (HR = 1.47, 95% CI: 0.71–3.05) (47). Figure 5 is a bar diagram representing minimum lymph node yield to be predictive of outcome of urinary bladder cancer as stated in different studies.

Prior to the publication of the LEA trial, majority of the retrospective and prospective literature were in favor of extended lymphadenectomy providing a survival advantage over standard lymphadenectomy. Many authors used factors like lymph node number, lymph density, extra nodal extension, etc., as markers of adequacy of lymphadenectomy and many studies showed significant correlation between number of lymph nodes dissected, lymph node density and oncological outcomes after radical cystectomy. Since extended lymphadenectomy was thought to harvest more lymph nodes and provide a better idea about lymph density it was recommended over and above standard dissection in these studies. Now, whether template is more important or number of harvested lymph nodes? A meticulous dissection with emphasis on skeletonization of the pelvic vessels is more appropriate than relying on the absolute lymph node count. There is a reasonable amount of consensus on this and that is why contemporary studies comparing various types of pelvic lymphadenectomy in bladder cancer make use of fixed anatomical templates rather than absolute number of lymph nodes.

### Current Guidelines

Both the EAU and AUA guidelines recommend PLND during RC. However, they are guarded in elaborating the extent of lymphadenectomy required. The EAU guidelines state that “extended LND might have a therapeutic benefit compared to less extensive PLND, but due to bias, no firm conclusions can be drawn” (48). The NCCN guidelines recommend that bilateral PLND should be performed, with a minimum of common iliac, internal iliac, and obturator nodes excised (49). It also states that a more extensive PLND, which may include the common iliac and the lower para-aortic and paracaval nodes may be associated with better survival and lower local recurrence rates. Still, it stops short of recommending this type of PLND. The AUA guidelines state that a bilateral PLND should be performed in every surgery with a curative intent (grade B). The standard PLND template with a minimum total lymph node count of 12 should be included (50). Even though one prospective randomized study has been published, the lack of robust clinical data on the various extents of PLND probably limits the scope of recommendations in the above guidelines.

## Conclusion and Recommendations

None of the surrogate markers of adequacy of PLND, namely anatomical template, lymph node number or lymph node density have been recommended forthright due to the lack of robust prospective data. In even the most recent meta-analysis on this topic, most of the included studies were retrospective. The differences in use of neoadjuvant chemotherapy worldwide also make it difficult to compare the studies without introducing bias. The extent of lymphadenectomy was left at the surgeon's discretion in most retrospective studies, thereby lacking randomization. Therefore, all of these issues should be accounted for before attempting to compare the different extents of PLND, reported in different studies. Therefore, to facilitate the above, based on this review, we wish to emphasize upon and recommend the following points:

- Level of pelvic lymph nodes are used to denote specific anatomical locations of the pelvic lymph nodes. The extent of pelvic lymphadenectomy would be denoted by whether just one, two, or all three levels of lymph nodes have been dissected out during PLND.
- Anatomical templates of dissection have been defined in the preceding sections and this nomenclature should be strictly followed in all future trials to bring uniformity and facilitate comparison.
- Whenever possible, we should adhere to guidelines and try to do an extended PLND, especially whenever there is visible lymphadenopathy on exploration and imaging.
- Super-extended PLND appears to have no survival benefit over extended PLND and its use should be decided on a case to case basis.
- PLND should be deemed adequate if an anatomical template is followed and thorough dissection is done rather than relying on the number of harvested lymph nodes or the lymph node density. Anatomical recommendations of a particular template are favorable and generalizable to a wider clinical community. This obviates the reliance on histopathologist for lymph node identification and subsequent prognostication.

## Author Contributions

RJ and NS have contributed equally to writing and manuscript preparation of this work. RJ and APS have conceived the idea if the review and have done the search strategy and selection of the articles and have contributed equally to the process of review and critical analysis. GC and AS have helped equally in the selection of articles for review and in final revision and manuscript preparation. All authors contributed to the article and approved the submitted version.

## Conflict of Interest

The authors declare that the research was conducted in the absence of any commercial or financial relationships that could be construed as a potential conflict of interest.
